# Increased ambulance on-scene times but unaffected response times during the first wave of the COVID-19 pandemic in Southern Denmark

**DOI:** 10.1186/s12873-022-00623-7

**Published:** 2022-04-09

**Authors:** Jennifer Rosenkjær Eskol, Floor Dijkstra Zegers, Daniel Wittrock, Annmarie Touborg Lassen, Søren Mikkelsen

**Affiliations:** 1 Department of Anaesthesiology and Intensive Care Medicine, Odense University Hospital, Odense, Denmark; 2 Center for Clinical Epidemiology, Odense University Hospital, Odense, Denmark; 3 Research Unit of Clinical Epidemiology, Department of Clinical Research, University of Southern Denmark, Odense, Denmark; 4 The Prehospital Research Unit, Region of Southern Denmark, Odense University Hospital, Odense, Denmark; 5 Ambulance Syd, The Emergency Medical System of the Region of Southern Denmark, Odense, Denmark; 6 Emergency Medicine Research Unit, Odense University Hospital, Odense, Denmark; 7 Department of Regional Health Research, University of Southern Denmark, Odense, Denmark

## Abstract

**Background:**

During the first wave of the COVID-19 pandemic, a lockdown was imposed on the Danish society. Reports from other countries that were hit by the COVID-19 pandemic before Denmark instilled fear of flooding of the emergency departments. To mitigate this flooding, increased competencies were conveyed to the paramedics in the ambulances aiming to allow for a release of a higher number of patients prehospitally.

The increased competencies in the prehospital personnel were expected to increase the on-scene time and thus the total workload of the ambulances potentially resulting in delays in the acute care. We sought to elucidate the effects of the pandemic on the workload of the prehospital system during the first wave.

**Methods:**

This was a retrospective study using operational data from the regional emergency medical dispatch centre in the Region of Southern Denmark. We collected the number of ambulance runs, the response times, the on-scene times, and the mission outcome of all ambulance runs with lights and sirens in the Region of Southern Denmark during the first wave of the pandemic. We compared the numbers with a similar period in the year before.

**Results:**

Compared with the year before the pandemic we observed a 10.3% reduction in call volume and a corresponding reduction in the total number of missions with lights and sirens. We found an increase in on-scene times in both missions with patients conveyed to hospital (20.6 min vs. 18.7 min) and missions with non-conveyed patients (37.4 min versus 30.7 min). The response times were unaffected.

**Conclusion:**

The increased on-scene times of the ambulances may largely be attributed to time utilised to exert the increased competencies concerning treat-and-release of patients.. Despite an increased on-scene time of the ambulances, we believe that the combination of a reduction in the number of total missions and the existing capacity in the ambulance service in the Region of Southern Denmark nullified the prolongation of ambulance response times that was seen in other countries during the pandemic. This capacity allowed for time spent performing in-depth examinations of patients with the potential to be released at the scene.

## Introduction

The ongoing worldwide pandemic caused by the SARS-CoV-2 virus (COVID-19) reached Denmark in February 2020. Worldwide, the pandemic subjected the health care systems to massive pressure. In order to mitigate the effects of the pandemic, a national lockdown was imposed on Danish society on March 11th^,^ 2020. A plethora of measures was imposed in an attempt to reduce the spread of COVID-19. Like in other countries, the measures involved precautions against public assembly of crowds, the shutting down of bars and restaurants, an increased focus on hygiene, and, in general, more outspoken approaches to avoid inter-personal transfer of the virus [1, 2]. Among the precautions imposed were specific mandatory alterations of prehospital procedures, such as the donning of full-body protective gowns and surgical face masks. Furthermore, the prehospital disinfection protocol following patient contact was expanded.

Under normal circumstances, according to the Danish regulations, only very few patients with clearly defined and manageable conditions may be released by the ambulance personnel following treatment. An example of such a condition is hypoglycaemia in a patient with previously known diabetes mellitus or an acute heroin overdose in a known drug addict. In most other conditions, the ambulance personnel is required to transport the patient to a hospital for further evaluation.

A massive flooding of the emergency departments was, however, expected and with the aim of reducing this influx of patients and keeping patients not requiring a hospital visit at bay, several measures were made to increase the paramedics’ ability to release patients prehospitally following treatment.

Specifically, regional test units were implemented allowing for the paramedics to have patients tested for COVID-19 in their homes. This information could increase the foundation necessary for releasing the patients after treatment. Furthermore, the paramedics were encouraged to call for consultation by the telephone from the prehospital physicians applied in mobile emergency care units. These consultations over the telephone were aimed at ensuring that patients with lesser conditions might safely be released at the scene following treatment by the paramedic alone.

In contrast to what was expected, almost immediately following the implementation of the lockdown, a notion occurred among the various branches of the Danish emergency medical system (EMS) that the number of patients contacting the EMS declined.

This notion is well supported by the international literature [3–7]. It is unknown whether this presumed reduction in the number of tasks did indeed occur in Denmark and whether any changes in the prehospital contact pattern influenced system variables such as ambulance response time and time spent at the scene in the EMS. Systematically gathered knowledge of the extent and the type of impact an epidemic may exert on prehospital transport may aid in planning for possible future events.

This study is an investigation of the changes in the amount of EMS contacts in the Region of Southern Denmark in the first 15 weeks of the lockdown compared with the identical period the year before the occurrence of the COVID-19 pandemic. Furthermore, we attempted to evaluate whether the EMS was burdened beyond its limits. As a surrogate marker for this measure, we investigated whether any changes in the response times of the ambulances dispatched with sirens and light occurred.

## Methods

### System setting

Denmark covers 43,000 km^2^ and is populated by 5,800,000 inhabitants. Denmark is divided into five health regions each responsible for the provision of healthcare in the region. This investigation concerned the EMS in the Region of Southern Denmark. This region comprises 1.2 million inhabitants distributed on 12,256 km^2^, roughly 20% of the Danish population [8]. The Danish prehospital emergency medical system is a publicly funded, nationally implemented system providing the same tax-funded prehospital basic service to every citizen in Denmark [9].

The prehospital system is a three-tiered system in which the basic resource is an ambulance manned with two emergency medical technicians (EMTs) [10, 11]. The prehospital units are dispatched by one of five emergency medical dispatch centres, one in each health region. The dispatcher, usually a nurse or a paramedic (PM), dispatches one of the following: an ambulance, an ambulance and a paramedic, or an ambulance and an anaesthesiologist-manned mobile emergency unit which can be either a ground-based unit (MECU) or one of four helicopters [helicopter emergency medical service (HEMS)]. In total, the Region of Southern Denmark has 70 ambulances, six MECUs at the disposal as well as shared use of the four helicopters in the HEMS. The dispatcher uses a criterion-based tool to assess the severity of the mission and to decide which tiers to activate. This criterion-based decision support tool has been in place since 2011 [12]. The mission characteristics (response time, time spent at the scene, mission outcome), as well as patient variables, are entered into a national electronic patient medical record system [13]. Apart from missions requiring immediate dispatch of ambulances, second and third-tier responses, the ambulances are used in missions of lesser acuity [12] and even as transport vehicles in interhospital transfers of patients.

### Study design

This study is a retrospective observational study of prospectively entered data.

### Data acquisition

We acquired prospectively registered operational data from the electronic patient medical record system and the dispatch system at the emergency medical dispatch centre in the Region of Southern Denmark. The data obtained did not include any information regarding the patients or the individual EMTs or PMs but included only data regarding the response times of the ambulances and the on-scene time of the ambulances. We studied the period of lockdown and a similar period one year before the lockdown. We thus compared the operational data concerning the weeks 11–25 in the year 2020 (the period defining the first wave of the COVID-19 epidemic in Denmark) with weeks 11–25 in the year 2019.

We included all missions in which an ambulance was dispatched with lights and sirens. For each mission, we registered the response time of the ambulance and the time spent at the scene by the ambulance personnel. In our analyses of time spent at the scene, we distinguished between missions in which the patient was admitted to the hospital and missions in which the patient was released at the scene. The time spent at the scene was calculated as the difference in the timestamps recorded when the ambulance reached the prehospital scene and when the transport of the patient was initiated. For non-conveyed patients, time at the scene was calculated as the difference in the timestamps recorded when the ambulance reached the prehospital scene, and the emergency medical technicians reported the ambulance available again. We further recorded the total on-scene time of all ambulances dispatched with lights and sirens in the two periods regardless of mission outcome. All the time indications were reported in minutes.

### Descriptive analysis and statistical methods

Data are presented as medians and quartiles. All data were analysed using non-parametric statistics (the Chi-square test or the Kruskal-Wallis tests for equality in populations, and Wilcoxon rank-sum test to compare response times in the two different periods). Differences were considered significant when *p* < 0.05. Data were analysed using R 4.0.3 (The R Foundation for Statistical Computing, Vienna, Austria [14] and STATA 17.0 (StataCorp, College Station, Texas, USA).

### Ethical approvals

As only operational data were studied, no person-identifiable data were accessed or used. Thus, according to the Danish Legislation, no scientific ethical approvals were required. The handling of data was thus carried out in strict accordance with the regional and national legislation.

### Consent for publication

Not applicable.

## Results

Overall, from March 12th through June 21st 2020 which encompasses the weeks 11–25 (the first wave of the COVID-19 pandemic in Denmark) the total call volume of the emergency medical dispatch centre in the Region of Southern Denmark decreased by 10.3% from 29,745 calls in 2019 to 26,683 calls in 2020. In the weeks 11–25 in 2019 (March 11th – June 23rd), the calls resulted in the dispatch of an ambulance 15,678 times. In weeks 11–25 in 2020, the calls to the emergency medical dispatch centre resulted in the dispatch of 13,989 ambulances. This corresponds to a 10.4% reduction in the number of patients treated. Of these patients, 83.7% were transported to a hospital during the COVID-epidemic while 86.2% were transported to the hospital the year before the epidemic. This difference was significant (*p* < 0.001). Thus, a greater proportion and a greater absolute number of patients were released at the scene following treatment in 2020 compared to 2019. The time spent at the scene by ambulances dispatched with lights and sirens differed between the two periods. In 2020, for each ambulance run, significantly more time was spent at the scene than in 2019 (*p* < 0.001) (Table 1). In spite of fewer ambulance runs during the initial wave of the pandemic, the total time spent at the scene by ambulances dispatched with lights and sirens during the weeks 11–25 in 2020 was 383,604 min compared to 364,605 in 2019. This corresponds to a 5.2% increase (*p* < 0.001). There was no difference in the response times of the ambulances in the two periods (*p* = 0.98). The median response time thus was 7.0 min in both periods. For details, see Table 1.

**Table 1:** The total call volume, number of patients, the mission outcome, the expenditure of time in all missions with and without transport of patients, and the response times during the 15 weeks of the initial wave of the COVID-19 pandemic in the Region of Southern Denmark compared with the same period the year before the pandemic. All ambulances dispatched with sirens and lights entered into the analyses. Data presented as numbers and percentages or minutes and quartiles

	2019	2020	***p***-value
**Total call volume**	29,745	26,683	N/A
**Total number of patients attended to with ambulances with sirens and lights**	15,678	13,989	N/A
**Number of patients released at the scene**	2160 (13.8%)	2274 (16.3%)	< 0.001
**Number of patients transported to hospital**	13,518 (86.2%)	11,715 (83.7%)	
**Minutes spent at the scene (released patients)**	30.7 (19.8–44.3)	37.4 (25.9–51.7)	< 0.001
**Minutes spent at the scene (transported patients)**	18.7 (13.7–24.8)	20.6 (14.8–27.5)	< 0.001
**Response time in minutes**	7.0 (4.8–10.3)	7.0 (4.8–10.2)	0.98

## Discussion

We observed a 10.3% reduction in call volume and a corresponding reduction in the total number of ambulances dispatched with lights and sirens in the Region of Southern Denmark during the first wave of the COVID-19 epidemic compared with the year before the pandemic. The time spent at the scene treating patients that were released following treatment increased by approximately 20% during the COVID-19 epidemic compared with the year before. The time spent at the scene in patients transported to hospitals also increased but only by 10% compared with the year before. These changes did not cause any changes in the response times.

Like our findings, a reduction in the number of EMS missions during the COVID-19 pandemic (and correspondingly in the number of visits to the emergency departments) has been described by other authors [3–7]. Many different explanations have been proposed: Lifestyle changes brought about by the imposed lockdown may have influenced the propensity to become injured in driving accidents or other recreational activities [15–19]. It is also possible that the fear of contagion of SARS-CoV-2 has reduced the incentive to call for emergency medical assistance [6]. It has even been reported that patients have refrained from calling on the health care system for fear of “disturbing” the system during the pandemic [20]. The decrease in EMS activations is likely not entirely explained by societal changes implemented in response to COVID-19, but also in the public’s perception of the workload within the hospitals, which may have led to behavioural changes. The observed decrease in the present study may be explained by the shutting down of almost all of the Danish society that in itself might have led to a reduction in the number of traffic accidents, a reduction in the number of domestic accidents, and a reduction in the number of incidents related to the gathering of people. Other possible reasons may be that patients could be avoiding the EMS for fear of acquiring a SARS-CoV-2 infection.

In contrast to the present study and other related studies, a study from New York reported a significant increase in demands on the EMS resources of 60% compared with the same period the year before the pandemic. The majority of this increase was the result of an increase in calls regarding respiratory or cardiovascular cases [21].

The differences between the findings in our study and similar studies and the findings in the study by Prezant et al. [21] may reflect organisational differences in the public’s factual or perceived access to acute health care. In Denmark, health care is funded by taxes and is without immediate costs to the patient. This applies to both visits at the general practitioner and acute hospital care. Thus, in general, there probably is overall confidence that sufficient emergency and non-emergency services exist and alternative resources for low acuity conditions (primary care physicians, other healthcare clinicians) are trusted even during a pandemic. Thus, it is probable that in Denmark, the emergency medical dispatch system was not used as a first choice in low-acuity situations.

The overall findings that the on-scene time was prolonged have been reported by other ambulance services during the COVID-19 pandemic [22–24]. The interim guidelines issued by the World Health Organization in March 2020 stated that personal protective equipment (medical masks, gowns, gloves, and eye protection) (PPE) should be applied when transporting suspected COVID-19 patients to the hospital [25]. In the Region of Southern Denmark, these guidelines were adopted by the ambulance services and consequently, the time spent applying PPE necessarily was added to the patient access interval or the on-scene time. The increase in patients released at the scene and the longer time spent at the scene for these patients was probably the result of a deliberate assignment of increased competencies to the ambulance personnel in evaluating patients and deciding who might be released at the scene following prehospital treatment. Thus, to reduce crowding at the emergency departments, specific prehospital units manned with experienced paramedics were established with the aim of performing an in-depth evaluation of the patient followed by consultations with in-hospital physicians or prehospital physicians regarding the appropriateness of leaving some patients at home. Similar systems were developed in other emergency medical systems resulting in an increased number of prehospital patients that could be released at the scene following treatment [5].

In contrast with other studies that reported prolonged response times during the COVID-19 epidemic [21, 24, 26], the EMS in the Region of Southern Denmark managed to retain a normal response time for ambulances during the first wave of the COVID-19 pandemic.

The number of ambulances available in the Region of Southern Denmark did not change between the two periods. Nor did the dispatch practices change. Thus, in all other respects than increased competencies of the paramedics and the incentives made for the prehospital personnel to either release patients at the scene based on the examinations and tests performed by the paramedics or based on consultations with in-hospital or prehospital physicians, the system remained static during the two observational periods.

Should the EMS have had less buffering capacity, an increased workload in an otherwise static system would lead to increased response times. The fact that no increase in response times was observed despite an increase in on-scene time may thus be attributed to a combination of the reduced demand for ambulances combined with the existence of a system with an acceptable buffering capacity.

### Limitations

One major limitation in this study is that only operational data were analysed. No person-identifiable data were investigated. As such, no measures of the distribution of diagnoses can be made, just as no discussion regarding mortality within specific ICD-10 classification diagnosis chapters can be made. Thus, the study does not address the nature of the missions nor the individual events that led to a citizen requesting an ambulance. In contrast to the data regarding ambulance runs, the data on call volume could not be obtained on a weekly basis rather were collected monthly. Thus, data on call volumes are slightly less accurate than data on ambulance runs.

A further limitation to this study is that it did not investigate the impact of the COVID-pandemic on the emergency departments. This has, however, been done previously on a Danish national basis, where the governmental national ‘shelter at home’ order was associated with a marked reduction in unplanned hospital attendances [27].

### Strengths

The strengths of this study lie in the organisation of the prehospital services in the Region of Southern Denmark. The dispatch of all the ambulances in the region is carried out from one dispatch centre. As no ambulances can be dispatched in the region unless dispatched by the emergency medical dispatch centre, data completeness is assured.

## Conclusion

The COVID-19 pandemic resulted in a reduction in call volume to the emergency medical dispatch centre and a reduction in the number of ambulances dispatched. The time spent at the scene was significantly increased as was the number of patients released following treatment. This increased time at the scene was probably the combined result of the application of PPE and the implementation of prehospital measures to treat and release more patients at home. Despite this increase in time spent at the scene, no increase in response times was recorded. We believe that the combination of a reduction in the number of missions combined with the capacity of the ambulance service in the Region of Southern Denmark probably nullified the prolongation of ambulance response times that was seen in other countries. Thus, we found that there was a capacity in the EMS to allow for more elaborate investigations performed by the EMS personnel with the aim of releasing a larger number of patients at the scene following prehospital treatment.

## Data Availability

The datasets generated and/or analysed during the current study are publicly available as a supplemental file. For interpretation of headings in the dataset please contact the corresponding author on reasonable request.
